# Motif mismatches in microsatellites: insights from genome-wide investigation among 20 insect species

**DOI:** 10.1093/dnares/dsu036

**Published:** 2014-11-06

**Authors:** Susanta K. Behura, David W. Severson

**Affiliations:** Eck Institute for Global Health and Department of Biological Sciences, University of Notre Dame, Notre Dame, IN 46556, USA

**Keywords:** microsatellite, insect genomes, single-nucleotide polymorphism, imperfect motif, simple sequence repeat

## Abstract

We present a detailed genome-wide comparative study of motif mismatches of microsatellites among 20 insect species representing five taxonomic orders. The results show that varying proportions (∼15–46%) of microsatellites identified in these species are imperfect in motif structure, and that they also vary in chromosomal distribution within genomes. It was observed that the genomic abundance of imperfect repeats is significantly associated with the length and number of motif mismatches of microsatellites. Furthermore, microsatellites with a higher number of mismatches tend to have lower abundance in the genome, suggesting that sequence heterogeneity of repeat motifs is a key determinant of genomic abundance of microsatellites. This relationship seems to be a general feature of microsatellites even in unrelated species such as yeast, roundworm, mouse and human. We provide a mechanistic explanation of the evolutionary link between motif heterogeneity and genomic abundance of microsatellites by examining the patterns of motif mismatches and allele sequences of single-nucleotide polymorphisms identified within microsatellite loci. Using Drosophila Reference Genetic Panel data, we further show that pattern of allelic variation modulates motif heterogeneity of microsatellites, and provide estimates of allele age of specific imperfect microsatellites found within protein-coding genes.

## 1. Introduction

Microsatellites, also known as simple sequence repeats, are tandem repeats of 1–6 bp motifs, which are ubiquitously found in eukaryotic genomes. One of the characteristic features of microsatellites is that they undergo rapid expansion or contraction in repeat number, leading to extensive length polymorphisms.^1–5^ Replication slippage is considered as the major cause of variation of microsatellite repeats.^6–8^ Slippage error creates a loop in one of the strands that gives rise to an insertion or a deletion in the subsequent replications, depending on the strand specificity (replicating or template strand) of loop formation. This leads to an increase or decrease of repeat length of the microsatellite. Several models have been proposed that explain the mutation processes of microsatellites.^8–11^ While the stepwise mutation model^12^ suggests that change in microsatellite loci occurs by one repeat unit at a time, study^13^ also shows that differential changes in the repeat number of microsatellites are associated with distinct probability under different mutational processes and demographic histories.

It is well known that microsatellite loci are associated with an elevated rate of mutation that leads to abundant sequence polymorphisms of the repeats.^14,15^ Mutation in specific microsatellite loci often creates imperfect motifs,^16^ and they are generally referred to as imperfect microsatellites. Studies have shown that intra-allelic variability of microsatellites is significantly associated with selective force that maintains microsatellite loci in genome.^17–21^ In spite of the known facts that repeat variation of microsatellite loci is critical for their maintenance in the genome and in some cases may have functional consequences,^18,22^ our understanding of motif imperfection in microsatellites is very limited.^11^ In this study, we aim at understanding genome-wide patterns of motif mismatches among different insect species (*n* = 20) with a primary objective of investigating the relationship between microsatellite length and motif mismatches, and its role in genomic abundance of imperfect microsatellites across species.

## 2. Materials and methods

### 2.1. Genome sequences

The genome sequences of insects investigated in this study include 15 species of order Diptera [12 Drosophilidae species (*D. melanogaster*, *D. simulans*, *D. sechellia*, *D. yakuba*, *D. erecta*, *D. ananassae*, *D. pseudoobscura*, *D. persimilis*, *D. willistoni*, *D. grimshawi*, *D. virilis*, and *D. mojavensis*) and 3 Culicidae species: *Aedes aegypti*, *Anopheles gambiae* and *Culex quinquefasciatus*], 2 species of Hymenoptera (*Nasonia vitripennis* and *Apis mellifera*) and 1 species each of Lepidoptera (*Bombyx mori*), Coleoptera (*Tribolium castaneum*) and Hemiptera (*Acyrthosiphon pisum*). Moreover, we also selected a few non-insect species, such as the budding yeast (*Saccharomyces cerevisiae*), the roundworm (*Caenorhabditis elegans*) and the mammalian genomes of mouse (*Mus musculus*) and human (*Homo sapiens*) for specific comparative analyses. In addition, we analysed chromosome X sequences of 29 inbred lines of *D. melanogaster* from the DGRP (Drosophila Genetic Reference Panel; http://dgrp.gnets.ncsu.edu/) to investigate population-level sequence variation of imperfect microsatellites.

The genome sequences of *A. aegypti* (AaegL1), *A. gambiae* (AgamP3) and *C. quinquefasciatus* (CpipJ1) were downloaded from https://www.vectorbase.org/. The genome sequences of the 12 *Drosophila* species were obtained from FlyBase (www.flybase.org). The assembly version of most of the 12 *Drosophila* genomes was r1.3 except *D. melanogaster* (r5.27), *D. pseudoobscura* (r2.10) and *D. virilis* (r1.2). The wasp (Nvit_1.0) and honey bee genome (Amel_2.0) sequences were downloaded from http://hymenopteragenome.org/. The aphid (v2.0), silkworm (v2.0) and beetle genome (tacs_superscaffolds v4.0) were obtained from http://www.aphidbase.com/, ftp://silkdb.org/pub/current/Genome/ and ftp://ftp.bioinformatics.ksu.edu/, respectively. The four non-insect genome sequences were downloaded from the UCSC genome server (ftp://hgdownload.cse.ucsc.edu/goldenPath/currentGenomes/). The assembly versions were yeast (SacCer_Apr2011), roundworm (WS220/ce10), mouse (GRCm38/mm10) and human (hg19, GRCh37). For the insect species, the names have been abbreviated as the first letter of the genus followed by three letters of the species name throughout the text and illustrations.

### 2.2. Identification of imperfect microsatellites

The imperfect microsatellites were identified from the genome assemblies using the SciRoKo 3.4 program, employing the approach that implemented fixed penalty motif mismatch criteria.^23^ The minimum score 15 and mismatch penalty 5 were used as search criteria to identify imperfect microsatellites of mono-, di-, tri-, tetra-, penta- and hexanucleotide repeats in each species. We also used slightly different parameters such as a minimum score of 15 and mismatch penalty of 3, and a minimum score of 10 and mismatch penalty of 5 to search microsatellites to investigate whether changes in parameter values affected the results of this study. In none of the searches, more than three consecutive mismatches per locus were allowed to identify the imperfect microsatellites.

### 2.3. Motif mismatches and microsatellite length

The number of imperfect microsatellites along with their length and number of mismatches of individual locus, as predicted by SciRoKo, were used for different comparative analyses among the species. The genomic abundance of imperfect microsatellites with different length and motif mismatches was chosen for different comparative analyses. First, we targeted microsatellites of length <30 bp for comparison between perfect (no mismatch) and imperfect repeats (each containing exactly one mismatch) in each genome. Here, microsatellites of length 20, 21, 22, 23 and 24 bp were identified in genome-wide manner in each species. Note that microsatellites of length <20 bp are not appropriate for this analysis as they are always perfect under our search conditions. Similarly, microsatellites above 25 bp in length may have two mismatches as per our search conditions. Second, we targeted longer microsatellites (35, 40, 45, 50, 55, 60, 65, 70, 75, 80 and 85 bp) to compare repeats that have different number (1, 2, 4 or 6) of mismatches. The third comparison was made between microsatellites that were at least 30 bp long and had <3 or ≥3 mismatches (note that microsatellites <30 bp canno't have more than three mismatches as per our search conditions). Selected non-insect species, such as the budding yeast (*S. cerevisiae*), the roundworm (*C. elegans*) and the mammalian genomes of mouse (*M. musculus*) and human (*H. sapiens*), were also analysed in genome-wide manner in a similar manner.

### 2.4. Generalized discriminant analysis between perfect and imperfect loci across species

The generalized discriminant analysis was performed using the principal coordinates method.^24^ The variation of frequency of perfect microsatellites and microsatellites that have same number of mismatches across the 20 species were decomposed into the principal coordinates. Then they were tested by permutation to determine if the variation was significant between perfect and imperfect repeats. First, distance matrices were generated separately from the frequency data of both groups of loci across species. They were then decomposed into component eigenvalues by the principal coordinate analysis. The canonical axes scores (position of multivariate points on the canonical axes) were then used to determine correlations between variables, and the statistical significance of the correlation was inferred from permutation procedure implemented in the program named ‘canonical analysis of principal coordinates’ (available at https://www.stat.auckland.ac.nz/~mja/Programs.htm).

### 2.5. Permutational analysis of variance and pair-wise a posteriori comparison

The permutational analysis of variance (PERMANOVA)^25^ was used to determine association between the number of mismatches and the length of microsatellites among the 20 species. The Euclidean distance was used as the measure of data variation. The pair-wise *a posteriori* comparisons were performed between different lengths of microsatellites (35, 40, 45, 50, 55, 60, 65, 70, 75, 80 and 85 bp containing 1, 2, 4 or 6 mismatches) among the 20 insect species as well as among the 4 non-insect species using PERMANOVA *t*-statistic. No data transformation or standardization was done prior to analysis. Unrestricted permutation of the data was allowed to perform the permutation tests (9,999 times) to determine the statistical significance of the analysis. The *P*-value of <0.05 was considered as the threshold of significance, unless stated otherwise.

### 2.6. Identification and analysis of single-nucleotide polymorphisms localized within imperfect microsatellites

We identified single-nucleotide polymorphisms (SNPs) that are localized within microsatellite loci in *D. melanogaster* and *A. gambiae* from the NCBI SNP database (ftp://ftp.ncbi.nlm.nih.gov/snp/). The microsatellites were partitioned to either as M_1_ types if the SNP alleles matched to the imperfect motif sequences or as M_0_ types if SNP alleles did not match to the motif sequences. To determine the significance of association of M_0_/M_1_ allelic patterns with variation of motif mismatches of imperfect microsatellites, microsatellites containing either <3 mismatches or ≥3 mismatches were determined in each species with repeat length being controlled (≥30 bp) for each locus. The count data of these four groups of imperfect microsatellites were used in 2 × 2 contingency tests (Yates' chi-square) to determine statistical significance of association between allelic variation and motif imperfection of microsatellites.

### 2.7. Analysis of imperfect microsatellites from DGRP data

To further investigate the role of mutations in motif mismatches and to estimate the allele age of imperfect microsatellite loci, we analysed genome sequences of *D. melanogaster* inbred lines from the DGRP data.^26^ We restricted this analysis only to microsatellite sequences of chromosome X among 29 randomly selected inbred lines. A comprehensive analysis of the microsatellites in the entire DGRP was not an aim of the present study because such a study has been performed earlier.^27^ Mutations in the microsatellites among the inbred lines were identified by sequence alignment with the reference genome. The imperfect microsatellite data (from DGRP lines) were used as a training dataset to classify the data of imperfect microsatellites generated from the dbSNP by linear regression using *Weka Explorer*.^28^

The phylogenetic analyses were conducted using *D. melanogaster* imperfect microsatellite loci from different inbred lines. The sequence of each locus was extended up to 1 kb of both ends of microsatellite and the extended sequences were used to calculate evolutionary distances among the inbred lines using the maximum composite likelihood implemented in MEGA4.^29^ The neighbor-joining method implemented in MEGA4 was used for constructing phylogenetic trees. The total number of mutations, number of polymorphic sites, average number of nucleotide differences and the number of fixed mutations between inbred lines were calculated by using DnaSP v 5.10.^30^ The allele age of microsatellite SNPs was calculated based on allele frequency and polymorphisms among the inbred lines in the extended sequences of microsatellites by the coalescence method.^17^ The coalescence times were predicted by using a maximum likelihood approach implemented in the algorithm ‘bdmc21’ (available at http://www.rannala.org/).

## 3. Results

### 3.1. Frequency of imperfect microsatellites in insect genomes

**Figure 1.**
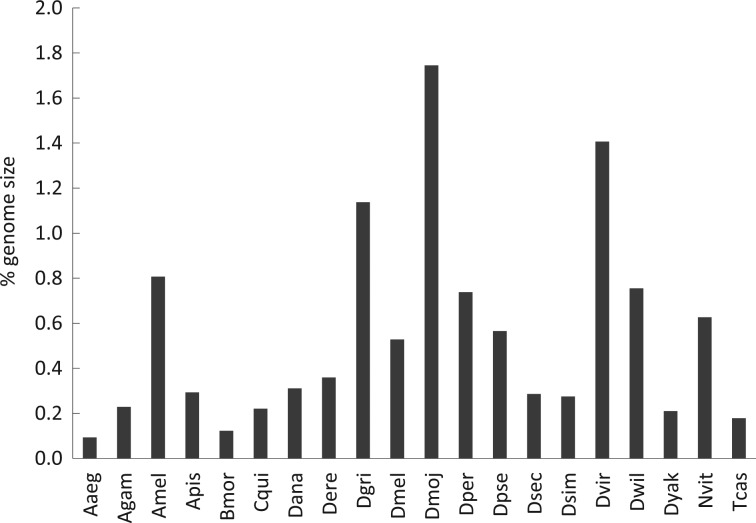
Imperfect microsatellites as the percentage of genome size of insects. The insect names (abbreviated) are shown in the *x*-axis and the percentages are shown in the *y*-axis.

The number of imperfect microsatellites varies among the insect species as given in Table 1. The imperfect repeats account to ∼15–46% of all simple sequence repeats identified from the genome sequences of the 20 insect species. The imperfect microsatellites represented <1% of the genome size of most of these insects except few *Drosophila* species (Fig. 1). The data in Table 1 and Fig. 1 show that species even within the Drosophila genus have extensive variation in the frequency of imperfect microsatellites. It was observed that specific *Drosophila* species (such as *D. mojavensis*, *D. virilis* and *D. grimshawi*) have a higher amount of imperfect microsatellites than other insects. Moreover, our data show that the imperfect microsatellites are differentially distributed among different chromosomes within the genome (Supplementary Table S1). This was evident in insects where chromosomes have been annotated from genome assemblies. It was observed that chromosome X harbours the highest proportion of imperfect microsatellites in *D. melanogaster*, a pattern that was also found in *D. simulans* and *D. yakuba*. However, this pattern is missing in non-*Drosophila* insects such as *A. gambiae* and *T. castaneum* (Supplementary Table S1). Furthermore, the proportion of imperfect microsatellites varies differentially among species depending on the motif length of microsatellites (Supplementary Table S2). The di- and trinucleotide repeats are associated with an elevated rate of motif mismatches compared with all other repeat sizes. Moreover, this pattern seems to be a conserved feature across the insect genomes.

**Table 1.** Number of imperfect microsatellites in different insect genomes

Species	Imperfect microsatellites	% of all microsatellites
Aaeg	22,620	15.5
Agam	30,207	28.1
Apis	44,563	24.1
Cqui	20,454	19.5
Dana	18,453	31.4
Dere	14,315	36.6
Dgri	74,637	46.1
Dmel	17,392	31.6
Dmoj	85,525	42.3
Dper	40,641	34.6
Dpse	36,289	33.6
Dsec	11,520	28.2
Dsim	10,995	30.0
Dvir	62,282	42.8
Dwil	55,121	35.6
Dyak	17,449	35.4
Amel	51,702	30.7
Nvit	28,074	23.4
Bmor	19,732	18.7
Tcas	5,155	28.8

The insect names are abbreviated with four letters; first letter represents the genus name and last three letters represent the species name. The percentage of microsatellites that are imperfect in the corresponding genome is also shown.

### 3.2. Motif mismatches and microsatellite length

**Figure 2.**
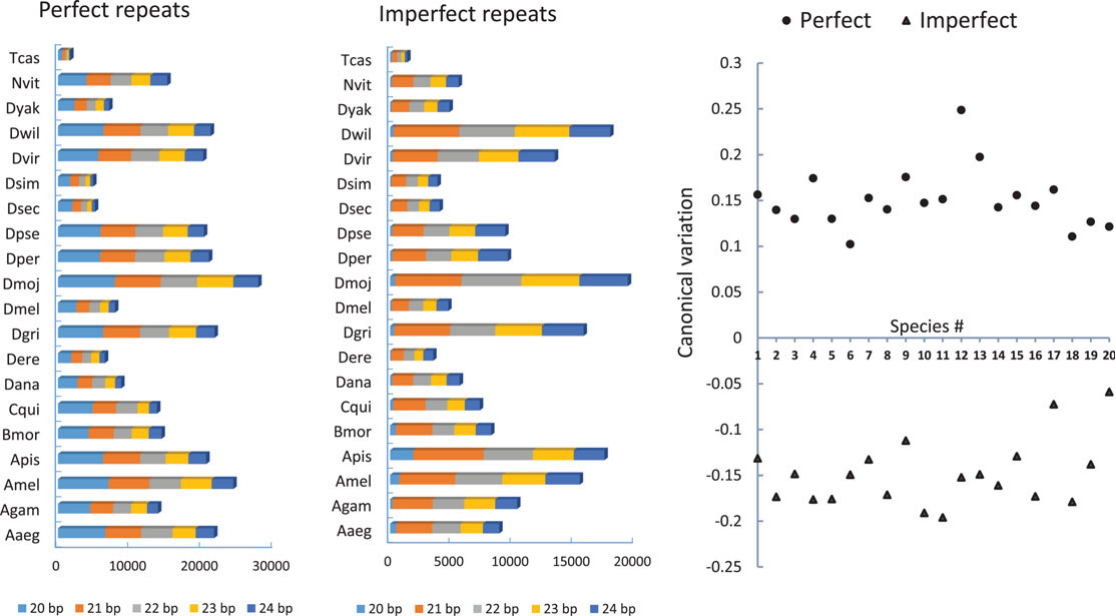
Stacked bar graphs showing variation in the number of perfect and imperfect repeats among the 20 insect species. The stacks in the graph represent different lengths of microsatellites as shown below the graphs. The scatter graph on the right shows the canonical variation of frequency of microsatellites of different length for each species. The species are indicated as 1 through 20 on *x*-axis and the canonical variation is shown in *y*-axis.

The mean length of imperfect microsatellites is higher than that of perfect microsatellites in each species (Supplementary Table S3). To understand the relationship between microsatellite length and motif imperfection, we identified microsatellites of different lengths (20, 21, 22, 23 and 24 bp) that either lack mismatch (perfect motifs) or have exactly one mismatch in each locus. The generalized discriminant analysis^24^ was conducted to determine the significance of variation between perfect and imperfect microsatellites across the genomes. Results of this analysis showed a significant relationship (first squared canonical correlation = 0.9024, *P* = 0.0001) between perfect and imperfect microsatellites among the species, wherein the imperfect microsatellites of different lengths revealed lower canonical variation than that of the perfect microsatellites in each species (Fig. 2).

Then to know if the extent of motif mismatches is related to microsatellite length, we determined the frequency of microsatellites of different lengths (35, 40, 45, 50, 55, 60, 65, 70, 75, 80 and 85 bp) harbouring different numbers of mismatches (1, 2, 4 and 6) in each of 20 species (Supplementary Table S4). The length and mismatch criteria were selected based on the search parameters as described above. The dataset was then used to perform permutational multifactorial analysis of variance of mismatch numbers and length of microsatellites. Results of this analysis showed that both the factors (length and mismatches) have significant association with the variation of genomic frequency of microsatellites among the species (*F* = 2.6, *P* = 0.02 for length and *F* = 45.02, *P* = 0.001 for mismatch). This suggested that genomic abundance of imperfect microsatellites may have an evolutionary link with the extent of sequence heterogeneity of repeat motifs across species. Moreover, it was also observed that genomic frequency of the microsatellites having only one mismatch correlates better with that of loci harbouring two mismatches than loci having four or six mismatches (Table 2). This again supported for a possible link between genomic frequency and extent of motif imperfection of microsatellites.

**Table 2.** Pearson correlation of frequency of imperfect microsatellites. The microsatellite lengths are shown in bp in the first column. The frequencies of microsatellites having 1 mismatch are compared with that of microsatellites having 2, 4 or 6 mismatches among the 20 insect species

Repeat length (bp)	Correlation (1 mismatch vs. 2 mismatches)	Correlation (1 mismatch vs. 4 mismatches)	Correlation (1 mismatch vs. 6 mismatches)
35	0.959	0.142	0.096
40	0.887	0.791	0.190
45	0.814	0.605	0.010
50	0.721	0.386	0.300
55	0.749	0.094	0.143
60	0.920	0.118	0.022
65	0.839	0.169	0.009
70	0.865	0.226	−0.136
75	0.967	0.361	0.054
80	0.939	0.692	0.271
85	0.756	0.474	0.430

### 3.3. Motif heterogeneity and genomic abundance of microsatellites

To further understand the relationship between motif heterogeneity and genomic abundance of microsatellites, we performed the following analyses. First, we calculated the expected number of mismatches in each imperfect microsatellite from the average rate of per-site mismatch (sum of mismatches divided by the sum of loci lengths) in individual genomes. Then for each imperfect microsatellite, the expected number of mismatches (repeat length multiplied with per-site mismatches) was calculated and compared with the observed number of mismatches in the loci. The results of this comparison showed that microsatellites that carry a higher amount of mismatches than expected are significantly related (Spearman *r* = 0.938, df = 19, *P* = 0.000001) with microsatellites that carry a lower amount of mismatches than expected across the species (Table 3). The former group of microsatellites consistently showed lower frequency in the genome than the latter group, indicating that genomic frequency of microsatellites is negatively influenced by motif heterogeneity of the repeats.

**Table 3.** Number of microsatellites where motif mismatches are either higher or lower than the expected values of mismatches in different insect genomes

Species	Higher than expected	Lower than expected
Aaeg	5,596	17,024
Agam	14,846	15,361
Apis	13,755	30,812
Cqui	6,525	13,929
Dana	5,766	12,687
Dere	5,322	8,993
Dgri	28,832	45,805
Dmel	7,945	9,447
Dmoj	34,735	50,790
Dper	15,174	25,467
Dpse	11,947	24,342
Dsec	4,116	7,404
Dsim	3,762	7,233
Dvir	23,855	38,427
Dwil	17,832	37,289
Dyak	6,386	11,063
Amel	17,893	33,809
Nvit	12,709	15,365
Bmor	5,443	14,289
Tcas	2,359	2,796

To further confirm that genomic abundance of microsatellites is associated with motif mismatches of microsatellites, we performed PERMANOVA^25^ test among different groups of motif mismatches (1, 2, 4 and 6 mismatches) of microsatellites among species (Supplementary Table S4). The results of this analysis revealed that the relationship between higher mismatches and lower genomic abundance of microsatellites is statistically significant (*P* = 0.0001). The pair-wise *a posteriori* comparison between microsatellites of different lengths showed that the significance level of association of genomic abundance varies differentially across the insect species based on the length of microsatellites (Supplementary Table S4). These results clearly suggested that microsatellite abundance in genome is intricately related to sequence imperfection of motifs and the length of the microsatellites across species.

We also compared microsatellites by controlling the length and number of motif mismatches of microsatellites across the species. Here, we restricted the comparison among loci that are at least 30 bp in length and have either <3 or ≥3 mismatches. These length and mismatch criteria were selected based on our search parameters as described in the Materials and methods section. The result of this analysis also revealed that loci that have less than three mismatches are more prevalent in the genome than those with three or more number of mismatches (Supplementary Table S5). This pattern was consistent not only in the insect species, but also in other unrelated species such as yeast, roundworm, mouse and human (Supplementary Table S5).

We also wanted to confirm that our observations were not influenced by the search parameters we used to identify the microsatellites. To test that, we used different parameters such as minimum score 15 and mismatch penalty 3 or minimum score of 10 and mismatch penalty 5 to search microsatellites in specific genomes. When data of these searches were used to repeat the analyses described above, we still found that microsatellites with higher mismatches tend to have lower prevalence in the genome (data not shown). This confirmed that the link between sequence imperfection and microsatellite abundance is a robust evolutionary process and is not influenced by the search parameters of microsatellites.

### 3.4. Mutation and imperfection of motif sequences

**Figure 3.**
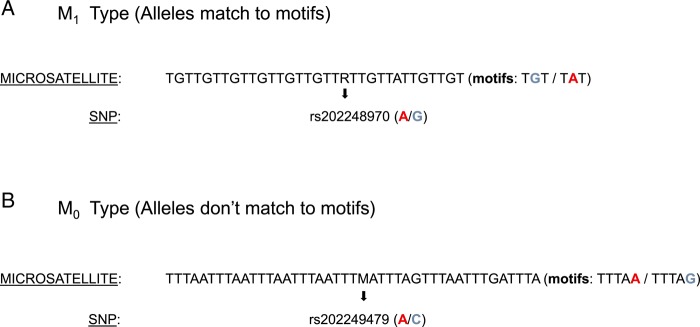
Imperfect microsatellites with M_1_ and M_0_ SNP alleles. (A) The M_1_ type: these are microsatellites where the SNP alleles match with the variant nucleotide of the motif sequences. The SNP ID is shown by rs # and the alleles are shown within parenthesis. The microsatellite and the mismatch motifs are shown, and both A and G are represented in the motifs. (B) The M_0_ type: these are microsatellites where the alternative SNP alleles are not found in the motif sequences of the microsatellite. Note that the C allele of the SNP is not represented in the mismatch motif.

To investigate role of mutation in motif imperfection, SNPs localized within microsatellite loci were identified from the dbSNP database (see Materials and methods). Then, we identified loci where motif mismatches corresponded to the alternate alleles of the cognate SNP (Fig. 3). These were referred to as M_1_ microsatellites. We also found microsatellites where motif mismatches did not correspond to the SNP alleles (Fig. 3). These were referred to as M_0_ microsatellites. The microsatellites with different numbers of mismatches (≥3 or <3 mismatches) and having either M_0_ or M_1_ allele patterns were identified in *D. melanogaster* and *A. gambiae* (Supplementary Table S6). The number of mismatches (≥3 or <3) was particularly chosen as 1) microsatellites <30 bp in length are predominant in both genomes, and 2) loci that are <30 bp have less than three mismatches as per our search conditions. The count data of microsatellites, based on the number of motif mismatches and pattern of allelic variation (M_0_ or M_1_), were analysed by Yates' chi-squared test. The result of this analysis revealed significant (*χ*^2^ = 315.5, *P* < 0.0001) association between allele sequences of SNPs and mismatch patterns of microsatellites in *D. melanogaster*. A similar result was observed in *A. gambiae* where the association was also statistically significant (*χ*^2^ = 13.6, *P* = 0.0002). Taken together, these findings show that patterns of allelic changes of microsatellites have a significant role in modulating the number of motif mismatches of imperfect microsatellites.

### 3.5. Insights from population data

**Figure 4.**
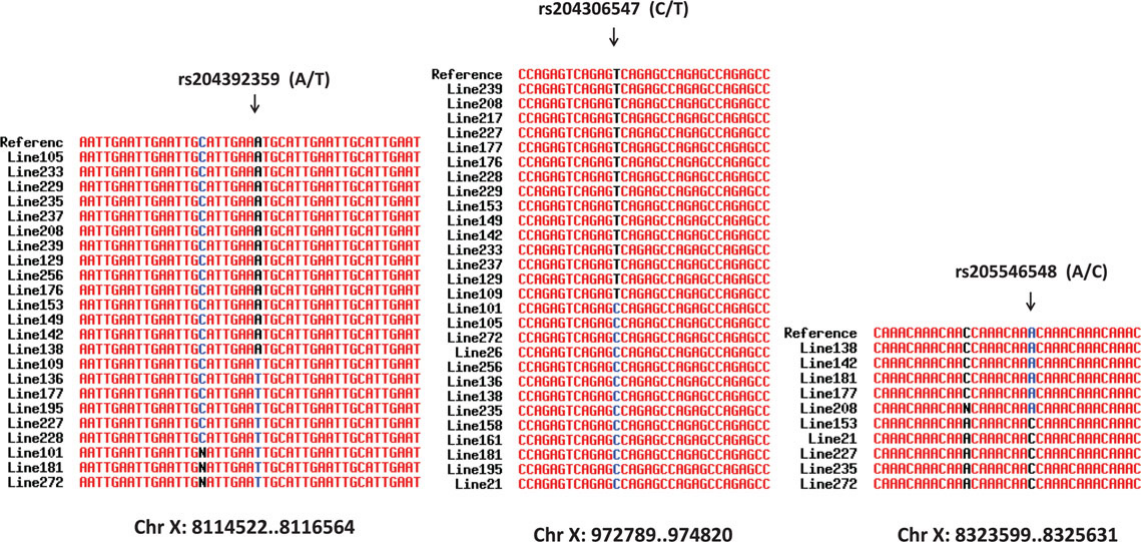
Multiple sequence alignment of microsatellite loci among different inbred lines of *D. melanogaster*. The chromosomal position of the microsatellite loci is shown below the alignment. The inbred line ID corresponding to each sequence is shown. The reference SNP identified in *D. melanogaster* (dbSNP data) is shown by a downward arrow along with SNP ID and alleles.

We also analysed sequences of imperfect microsatellites of multiple inbred lines (*n* = 29) of *D. melanogaster* (DGRP data) to understand within-species variation of motif sequences of imperfect microsatellite loci. We identified a total of 118,446 imperfect microsatellites from the chromosome X sequences of the 29 inbred lines of *D. melanogaster* (Supplementary Table S7). Of these, a total of 1,025 loci showed polymorphic microsatellites (Fig. 4). We used the allele sequences and motif mismatch patterns of these 1,025 microsatellites to train the imperfect microsatellites identified from dbSNP data. Linear regression-based data classification of SNP allelic variation (M_0_ versus M_1_) and the corresponding motif mismatch patterns of microsatellites were performed using Weka3.6.^28^ The results of these analyses revealed a significant relationship (*P* < 0.05) between allele variation and motif mismatches of imperfect microsatellites, and also showed that for accumulation of a single M_1_ type of mutation, the number of repeat mismatches in the microsatellite decreases by 56%. This further confirmed that pattern of allelic variation plays a major role in motif heterogeneity of microsatellites.

**Figure 5.**
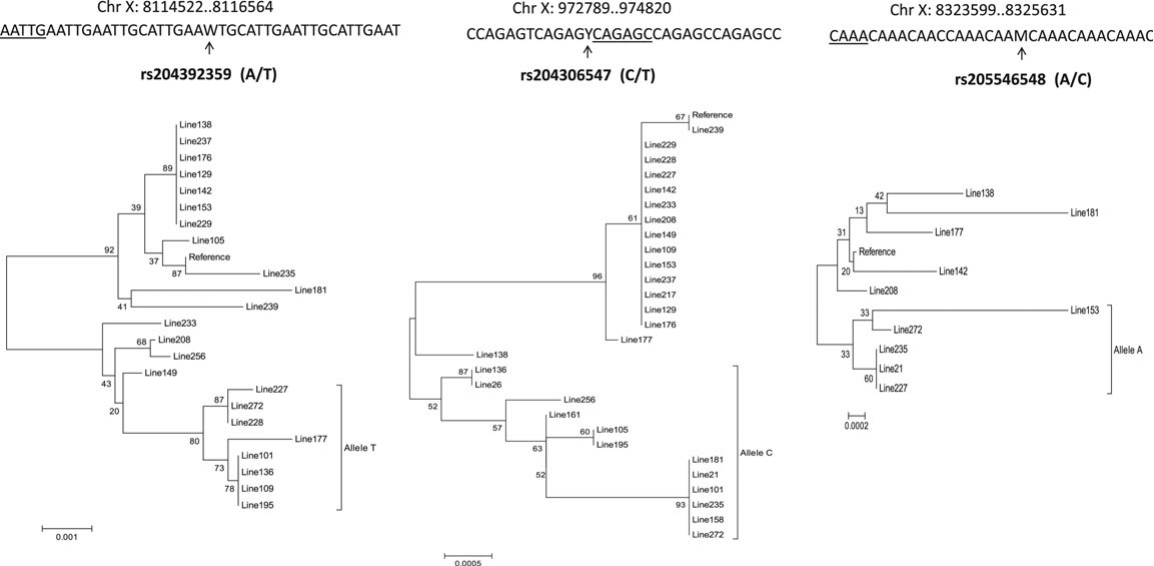
Neighbour-joining phylogenetic trees of microsatellites loci (with 1 kb flanking sequences of both ends) of *D. melanogaster* inbred lines. The chromosomal positions, the microsatellite repeat and the SNP position within the microsatellites (upward arrow) are shown on the top of each tree. The sequence origins (inbred line # or the reference genome) are also shown for each branch along with bootstrap support values. The phylogenetic grouping of *D. melanogaster* lines that carry the alternative allele of the SNP is also shown. The scale of branch length is shown below each tree.

**Figure 6.**
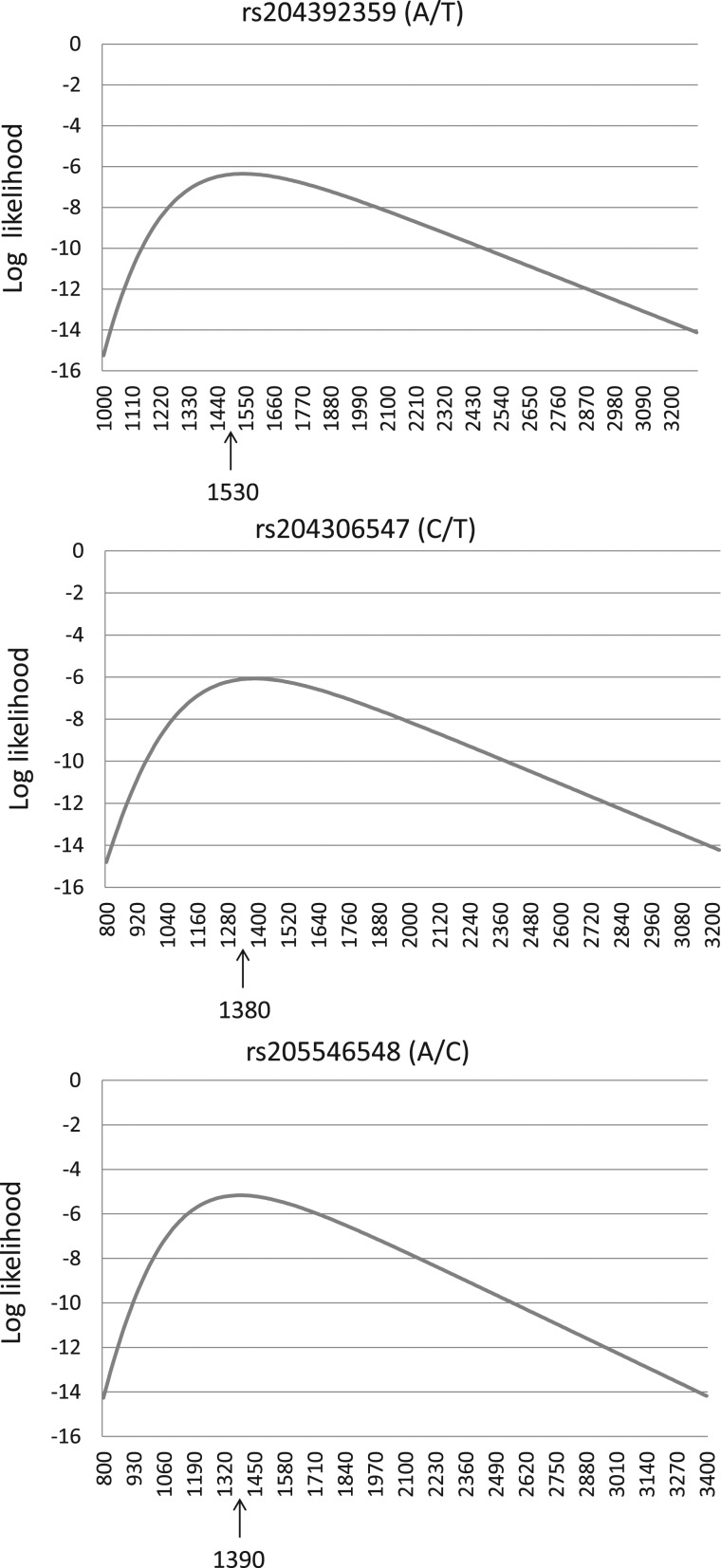
Distribution of likelihoods of allele age (generations ago) of the mutation (SNP) in the microsatellite sequences. The SNP IDs along with their alleles are shown above the graphs. The *x*-axis represents time (in generations) when the mutation likely occurred, and the corresponding estimates of likelihoods are shown in the *y*-axis. The specific generation time when the likelihood value is highest is indicated by an upward arrow.

We also estimated the age of specific alleles of imperfect microsatellites based on the population data (DGRP). First, we identified flanking mutations (within 1 kb on both ends) of microsatellites that were in complete linkage with segregating mutation within the microsatellite (Supplementary Fig. S1). Based on the results of phylogenetic analysis, the extended sequences showed differential genealogical relations among the inbred lines (Fig. 5). We determined the number of fixed mutations, total number of variable sites and the average number of nucleotide differences in the extended sequences of imperfect microsatellites (Supplementary Table S8). Based on the allele frequency of mutations within and the flanking sequences of the imperfect microsatellite, we predicted generation times that might have lapsed since the origin of the mutation in the microsatellite. The maximum likelihood method^17^ was used for making this prediction from the joint distribution of the number of copies in the population and the coalescence times of the intra-allelic variation. The results revealed that the maximum likelihoods varied between 1,380 and 1,530 generations (Fig. 6). Assuming 36 days as the average generation time of *D. melanogaster*, it was thus estimated that these alleles might have arisen 136–150 yrs ago.

## 4. Discussion

The results obtained from this study provide insights into sequence imperfection of microsatellite motifs in a genome-wide manner. We chose insect genomes for this investigation as microsatellites have been extensively exploited as molecular markers in ecology and population studies of numerous insect species,^31^ yet the imperfect microsatellites of insects remain poorly understood. As studies on evolution of microsatellites are gaining renewed attention with the advent of high-throughput DNA sequencing and whole-genome sequences,^32–39^ the present study was initiated as an effort to gain insights into the motif mismatch patterns of microsatellites of insects as well as specific non-insect eukaryotes. In this investigation, imperfect microsatellites were computationally identified from the draft genome sequences of the chosen species by SciRoko.^23^ Apart from SciRoKo, other computational algorithms such as IMEx, NWISL and Phobos are also available to identify imperfect microsatellites^40^ (http://ssr.nwisrl.ars.usda.gov/ and www.ruhr-uni-bochum.de/ecoevo/cm/cm_phobos.htm). However, comparing the efficacy of different algorithms in detecting imperfect microsatellites was not the aim of this study. We analysed each genome using the same tool (SciRoKo) and the same parameters appropriate for data comparison across genomes. The genome-wide search of microsatellites in insect species by SciRoKo revealed that the proportion of microsatellites with imperfect motifs varies from species to species even within the same genus. The 12 Drosophila species clearly represent that pattern (Table 1 and Fig. 1). Earlier study has shown that microsatellite abundance diverges rapidly between Drosophila species, and that considerable variation in the relative abundance of motif size classes exists between species.^41^ Moreover, differential association of microsatellites with coding sequences of Drosophila genome may impose differential constraint on microsatellite abundance between species. In fact, the number of microsatellites associated with coding sequences varies extensively among the 12 Drosophila genomes as shown from the previous study.^36^ At the same time, it is possible that the quality of genome assembly may affect identification of microsatellites comprehensively. However, the 20 insect genomes we analysed represented different qualities of genome assemblies of the insect species. Yet, our results showed highly consistent patterns in microsatellite imperfections across species (Supplementary Table S4), indicating that the results of this study are not affected by differences in assembly quality of the genomes.

We found that sex chromosomes tend to have elevated frequency of imperfect microsatellites than autosomes in *Drosophila* species. It is likely that differential association of microsatellites with coding sequences of different chromosomes may impose differential constraint on non-random prevalence of imperfect microsatellites within the genome. These coding repeats may be under differential selection pressure in the genome. It is known that repeats representing amino acid runs in *Drosophila* are under positive selection.^34^ Furthermore, in *D. melanogaster*, the adaptive evolution proceeds more quickly in X than autosomes.^42^ This is commonly referred to as the faster-X hypothesis. Such X bias adaptive evolution is associated with a number of phenotypic, morphological and behavioural changes in the fly.^43^ As sex chromosomes in *Drosophila* have elevated frequency of imperfect microsatellites than autosomes, the possibility of coding microsatellites playing a role in faster X evolution of Drosophila genome canno't be ruled out. Furthermore, our earlier study has shown that the trinucleotide repeats represent the highest fraction of codon repeats in insects.^36^ From the current study, we also observe that trinucleotide microsatellites show higher variation in motif imperfect than other motif sizes in several insects including *D. melanogaster* (Supplementary Table S2). It is likely that rapid variation of codon repeats in chromosome X may cause faster adaptive evolution of genes localized in X chromosome than autosomes. However, it is unlikely that such a mechanism is operational in all insect species. This is because no evidence of elevated frequency of imperfect microsatellites in chromosome X compared with autosomes was observed in the non-*Drosophila* species we investigated.

We further observed that microsatellite length and the extent of motif imperfection are significantly associated with the genomic abundance of imperfect microsatellites. Previously, it has been shown that longer microsatellites (>15 repeats) are associated with mutational bias that reduces the length of microsatellites.^44^ Hence, the older mutations are more likely to be deleted from the longer microsatellites, causing a paucity of long microsatellites in the genome. Although the length of microsatellites is known to have a role in the mutational activities of microsatellites,^45,46^ results from the current investigation show that sequence imperfection of motifs is a key determinant of abundance of imperfect microsatellites in the genome. Such an evolutionary link is not specific to insects, but is also observed in unrelated eukaryotes including yeast, worm and mammals. Consistent with this theory, the perfect microsatellites are found more abundantly than the imperfect microsatellites in each genome (Table 1). Moreover, it is known that mutations in microsatellite alleles shorter than a critical length favour repeat expansion, whereas mutations acting on longer alleles favour contraction of microsatellite repeats.^47^ The intricacies between length and sequence mismatches may also be critical in maintaining imperfect repeats in the genome.^21^ This is also indicated from comparison of genomic abundance of imperfect microsatellites among insect species based on different lengths of microsatellites (Supplementary Table S4). The posterior *t*-test *P*-values were significant for all the pair-wise comparisons among the 20 insect species. We, however, failed to observe such relationships among the non-insect species most probably due to the sample size (only four) used for analysis. Also, the large differences in the genome sequences of these species (worm, yeast, human and mouse) may account for the lack of significant relationships in imperfect microsatellites in the genomes.

Recently, many inbred lines of *D. melanogaster* were sequenced that generated a very useful resource of genetic variation data of fruit fly, and also provided new insights on the faster X hypothesis.^26^ We used these data to identify imperfect microsatellites in multiple inbred lines. The data were used to classify the reference SNP alleles (dbSNP data) of imperfect microsatellites to understand how mutation affects sequence imperfection in microsatellites. The results of that analysis suggested that there is a significant relationship between motif imperfection and mutational patterns in the microsatellite loci. Our data showed that the extent of motif heterogeneity of imperfect microsatellites is significantly linked to the nature of allelic changes in microsatellites. When a new allele is introduced that differs in sequence from that of existing mismatches in the microsatellite, the extent of sequence heterogeneity increases. On the other hand, when the introduced alleles match to the pre-existing motif mismatches, it does no't affect the motif heterogeneity for which the locus is more likely to be maintained in the genome than a locus where motif heterogeneity builds up by mutation. If mutations continue in a microsatellite wherein the introduced alleles build up motif heterogeneity, over time the locus eventually becomes a non-microsatellite sequence. Our result shows that, depending on the introduced allele sequences, a single mutation can reduce motif mismatches by 56%. It is plausible that this relationship may be instrumental in regulating the overall slippage events of simple sequence repeats in the genome.

In *D. melanogaster*, we further observed that imperfect microsatellites are also localized within specific protein-coding genes (Fig. 5). The microsatellite-associated SNP rs204392359 is localized in the intron of gene *stardust* (*sdt*). Similarly, the SNP rs205546548 is localized within a microsatellite in downstream region of gene *trf2* (TATA box-binding protein-related factor 2), and the SNP rs204306547 is localized within an intronic microsatellite of the gene *CG3655*. Furthermore, we estimated that alleles of these microsatellites originated within the last 150 yrs, suggesting that these imperfect microsatellites may be undergoing recent mutations in *D. melanogaster.* It is possible that these imperfect microsatellite and the associated mutations have a functional role in *D. melanogaster*, as increasing evidence now suggests that microsatellites are associated with functional and evolutionary roles in eukaryotic genomes.^14,48^ Moreover, in *A. gambiae* genome, numerous protein-coding genes harbour mutation in microsatellite sequences within the respective genes (Supplementary Table S9), indicating that motif imperfection of microsatellites may have extensive functional consequences in *A. gambiae*.

In conclusion, the current study is the first detailed investigation of imperfect microsatellites in insect species representing five taxonomic orders (Diptera, Hymenoptera, Lepidoptera, Coleoptera and Hemiptera). The results obtained from this investigation provide new insights into the evolution of sequence imperfection in microsatellite loci across genomes. The outcome of this study may have utility in assessing the role of motif imperfection of microsatellites in genome structure and function of insects.

## Supplementary data

Supplementary data are available at www.dnaresearch.oxfordjournals.org

## Funding

This work was supported from grants RO1-AI079125-A1, RO1-AI081795 and R21-AI101345 from the National Institute of Allergy and Infectious Diseases, National Institutes of Health, USA. Funding to pay the Open Access publication charges for this article was provided by the University of Notre Dame.

## Supplementary Material

Supplementary Data
