# Supplementary material for: Motif mismatches in microsatellites: insights from genome-wide investigation among 20 insect species
Source: DNA Res. 2014 Nov 6;22(1):29–38. doi: 10.1093/dnares/dsu036 (PMC4379975; doi:10.1093/dnares/dsu036)
Supplement: Supplementary Data [file supp_22_1_29__index.html]

Motif mismatches in microsatellites: insights from genome-wide investigation among 20 insect species — Supplementary Data 

# Motif mismatches in microsatellites: insights from genome-wide investigation among 20 insect species

## Supplementary Data

Supplementary Data

**Files in this Data Supplement:**

- Supplementary Figure 1 - tif file
- Supplementary Table 1 - docx file
- Supplementary Table 2 - docx file
- Supplementary Table 3 - docx file
- Supplementary Table 4 - docx file
- Supplementary Table 5 - docx file
- Supplementary Table 6 - xlsx file
- Supplementary Table 7 - docx file
- Supplementary Table 8 - docx file
- Supplementary Table 9 - xlsx file
