# Supplementary figures and images for: Motif mismatches in microsatellites: insights from genome-wide investigation among 20 insect species
Source: DNA Res. 2014 Nov 6;22(1):29–38. doi: 10.1093/dnares/dsu036 (PMC4379975; doi:10.1093/dnares/dsu036)

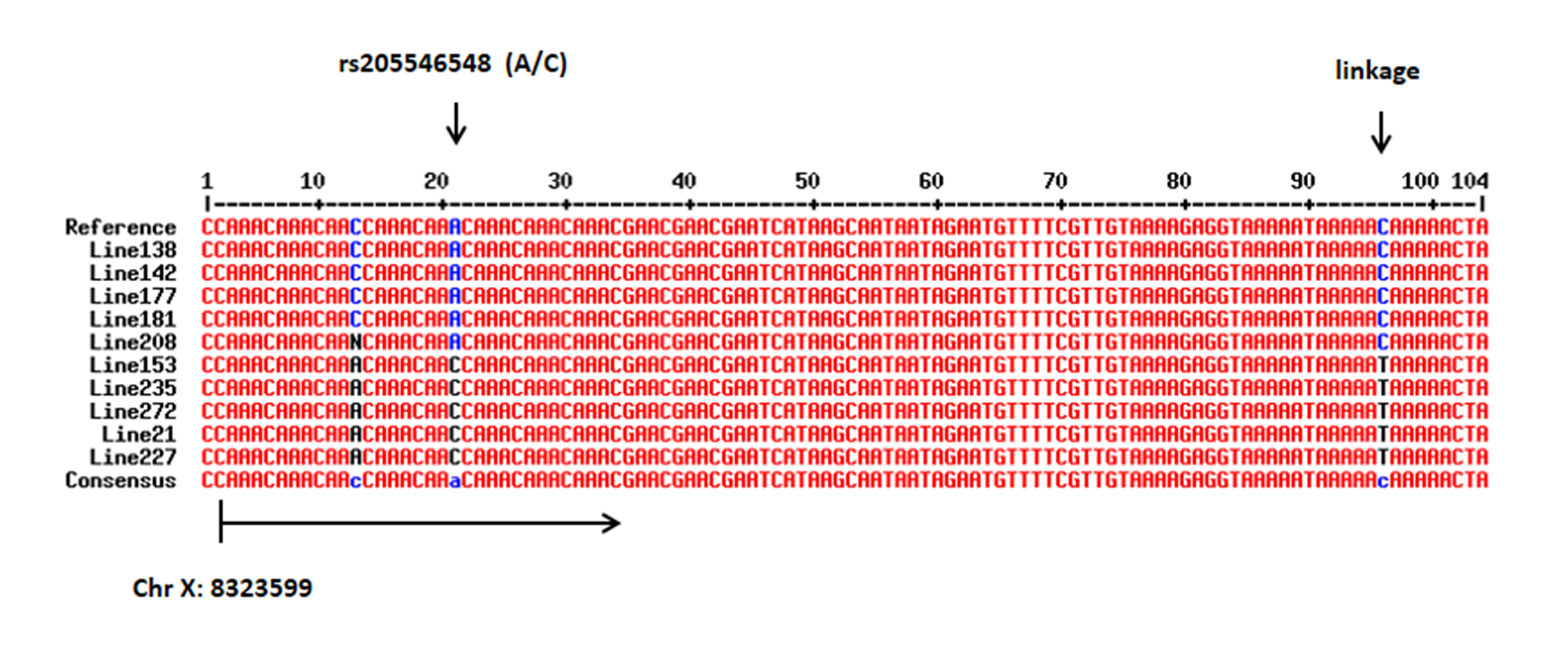

Supplement: Supplementary Data [file supp_dsu036_dsu036supp_figure1.tif]
