# Supplementary material for: Motif mismatches in microsatellites: insights from genome-wide investigation among 20 insect species
Source: DNA Res. 2014 Nov 6;22(1):29–38. doi: 10.1093/dnares/dsu036 (PMC4379975; doi:10.1093/dnares/dsu036)
Supplement: Supplementary Data [file supp_dsu036_dsu036supp_table1.docx]

Supplementary Table 1. Frequency of imperfect microsatellites in different chromosomes annotated from genome sequences of specific insects.

| Species/ chromosome* | Toal | Imperfect microsatellites | Percentage* |
| --- | --- | --- | --- |
| *Anopheles gambiae* | |  |  |
| 2R | 26704 | 7300 | 6.798793 |
| 2L | 21149 | 5654 | 5.265805 |
| X | 16956 | 5507 | 5.128898 |
| 3R | 20186 | 5395 | 5.024587 |
| 3L | 15366 | 4068 | 3.788697 |
| UNKN | 7001 | 2281 | 2.12439 |
| Y_unplaced | 10 | 2 | 0.001863 |
| *Drosophila melanogaster* | | |  |
| X | 14474 | 4701 | 8.538269 |
| Uextra | 7240 | 3181 | 5.777544 |
| 3R | 9191 | 2645 | 4.804025 |
| 3L | 7800 | 2117 | 3.845036 |
| 2R | 6815 | 1994 | 3.621635 |
| 2L | 6487 | 1782 | 3.236587 |
| U | 1601 | 560 | 1.017109 |
| 3RHet | 372 | 123 | 0.223401 |
| 2RHet | 373 | 90 | 0.163464 |
| 4 | 247 | 85 | 0.154383 |
| 3LHet | 328 | 83 | 0.15075 |
| XHet | 53 | 17 | 0.030877 |
| YHet | 42 | 8 | 0.01453 |
| 2LHet | 35 | 6 | 0.010898 |
| *Drosophila simulans* | | |  |
| X | 7134 | 2414 | 6.579449 |
| 3R | 7200 | 2145 | 5.84628 |
| 3L | 5705 | 1594 | 4.344508 |
| 2R | 5138 | 1525 | 4.156446 |
| 2L | 4615 | 1291 | 3.51867 |
| 4 | 125 | 36 | 0.098119 |
| *Drosophila yakuba* | |  |  |
| X | 13992 | 5420 | 10.99369 |
| 3R | 9270 | 3267 | 6.62664 |
| 3L | 7595 | 2599 | 5.271698 |
| 2R | 5998 | 2084 | 4.227095 |
| 2L | 5800 | 1962 | 3.979635 |
| 4 | 186 | 64 | 0.129815 |
| *Tribolium castaneum* | | |  |
| ChLG3 | 3196 | 806 | 4.503045 |
| ChLG9 | 1658 | 499 | 2.787865 |
| ChLG8 | 1648 | 474 | 2.648193 |
| ChLG6 | 1325 | 381 | 2.128611 |
| ChLG10 | 1255 | 375 | 2.095089 |
| ChLG5 | 1272 | 355 | 1.983351 |
| ChLG2 | 1137 | 325 | 1.815744 |
| ChLG7 | 1230 | 307 | 1.71518 |
| ChLG4 | 993 | 257 | 1.435834 |
| ChLGX | 560 | 131 | 0.731884 |

*Percentage is calculated based on total number of microsatellites in the genome, not based on individual chromosome.
