# Supplementary material for: Motif mismatches in microsatellites: insights from genome-wide investigation among 20 insect species
Source: DNA Res. 2014 Nov 6;22(1):29–38. doi: 10.1093/dnares/dsu036 (PMC4379975; doi:10.1093/dnares/dsu036)
Supplement: Supplementary Data [file supp_dsu036_dsu036supp_table2.docx]

Supplementary Table 2. Motif length and percentage of imperfection of microsatellites in different species.

| Species | Mono | Di | Tri | Tetra | Penta | Hexa |
| --- | --- | --- | --- | --- | --- | --- |
| Aaeg | 12.19 | 23.93 | 19.24 | 17.05 | 8.85 | 19.19 |
| Agam | 10.19 | 33.77 | 30.67 | 21.99 | 10.41 | 17.24 |
| Apis | 20.9 | 59.5 | 48.3 | 25.8 | 11.9 | 15.1 |
| Cqui | 16.8 | 33.29 | 15.34 | 24.52 | 9.37 | 21.23 |
| Dana | 25.8 | 38.11 | 38.27 | 40.42 | 14.21 | 28.98 |
| Dere | 24.54 | 47.59 | 47.13 | 36.04 | 21.34 | 32.9 |
| Dgri | 30.61 | 54.16 | 50.21 | 34.51 | 21.34 | 35.49 |
| Dmel | 16.17 | 36.16 | 42.48 | 28.24 | 31.67 | 29.44 |
| Dmoj | 21.78 | 51.18 | 47.96 | 41.22 | 22.8 | 38.51 |
| Dper | 20.69 | 38.35 | 40.53 | 49.32 | 17.71 | 33.2 |
| Dpse | 19.29 | 39.61 | 41.77 | 34.59 | 19.97 | 33.05 |
| Dsec | 18.6 | 37.29 | 42.56 | 26.86 | 15.08 | 25.94 |
| Dsim | 20.99 | 36.71 | 41.45 | 26.11 | 18.48 | 26.99 |
| Dvir | 22.54 | 48.72 | 46.06 | 49.42 | 18.44 | 35.87 |
| Dwil | 24.66 | 42.93 | 38.59 | 33.7 | 17.02 | 27.95 |
| Dyak | 20.73 | 45.85 | 46.83 | 36.07 | 20.16 | 32.03 |
| Amel | 28.25 | 34.85 | 36.42 | 26.48 | 20.23 | 27.5 |
| Nvit | 8.54 | 25.09 | 25.36 | 16.34 | 10.27 | 18.54 |
| Bmor | 14.59 | 31.33 | 19.51 | 20.55 | 14.58 | 15.52 |
| Tcas | 25.56 | 38.74 | 42.56 | 24.51 | 15.24 | 22.74 |
