# Supplementary material for: Motif mismatches in microsatellites: insights from genome-wide investigation among 20 insect species
Source: DNA Res. 2014 Nov 6;22(1):29–38. doi: 10.1093/dnares/dsu036 (PMC4379975; doi:10.1093/dnares/dsu036)
Supplement: Supplementary Data [file supp_dsu036_dsu036supp_table3.docx]

Supplementary Table 3. Average length of imperfect microsatellites compared to that of perfect microsatellites.

| Species | Perfect repeats | Imperfect  repeats |
| --- | --- | --- |
| Aaeg | 21 | 54 |
| Agam | 21 | 42 |
| Apis | 19 | 32 |
| Cqui | 21 | 58 |
| Dana | 19 | 36 |
| Dere | 19 | 36 |
| Dgri | 20 | 35 |
| Dmel | 21 | 55 |
| Dmoj | 21 | 37 |
| Dper | 20 | 32 |
| Dpse | 19 | 31 |
| Dsec | 18 | 39 |
| Dsim | 18 | 33 |
| Dvir | 21 | 43 |
| Dwil | 19 | 31 |
| Dyak | 19 | 34 |
| Amel | 20 | 34 |
| Nvit | 29 | 43 |
| Bmor | 19 | 33 |
| Tcas | 21 | 52 |
