# Supplementary material for: Motif mismatches in microsatellites: insights from genome-wide investigation among 20 insect species
Source: DNA Res. 2014 Nov 6;22(1):29–38. doi: 10.1093/dnares/dsu036 (PMC4379975; doi:10.1093/dnares/dsu036)
Supplement: Supplementary Data [file supp_dsu036_dsu036supp_table4.docx]

Supplementary Table 4. Genomic abundance of imperfect microsatellites of different lengths and pairwise a posteriori comparisons across species. Number of microsatellite loci in different species (name in first column) that have same length (header row) but varying number of mismatches (last column) are shown the data tables. The results of a posteriori comparisons are shown below the data tables.

*A)* Insect species: genomic abundance

| Species | **35 bp** | **40 bp** | **45 bp** | **50 bp** | **55 bp** | **60 bp** | **65 bp** | **70 bp** | **75 bp** | **80 bp** | **85 bp** | Number of mismatches |
| --- | --- | --- | --- | --- | --- | --- | --- | --- | --- | --- | --- | --- |
| Aaeg | 120 | 53 | 32 | 26 | 15 | 13 | 7 | 15 | 8 | 10 | 13 | 1 |
| Agam | 182 | 76 | 75 | 52 | 45 | 32 | 28 | 23 | 20 | 14 | 14 | 1 |
| Amel | 335 | 164 | 92 | 67 | 52 | 25 | 26 | 6 | 8 | 2 | 8 | 1 |
| Apis | 311 | 136 | 79 | 25 | 24 | 15 | 16 | 6 | 6 | 4 | 2 | 1 |
| Bmor | 141 | 29 | 8 | 2 | 2 | 3 | 1 | 1 | 3 | 3 | 2 | 1 |
| Cqui | 108 | 36 | 15 | 2 | 3 | 0 | 0 | 1 | 0 | 0 | 0 | 1 |
| Dana | 647 | 277 | 142 | 50 | 43 | 24 | 13 | 13 | 8 | 6 | 8 | 1 |
| Dere | 102 | 35 | 18 | 13 | 3 | 2 | 6 | 3 | 2 | 1 | 3 | 1 |
| Dgri | 712 | 232 | 107 | 90 | 46 | 29 | 19 | 14 | 8 | 12 | 10 | 1 |
| Dmel | 403 | 132 | 47 | 5 | 5 | 3 | 2 | 2 | 1 | 0 | 0 | 1 |
| Dmoj | 365 | 77 | 33 | 12 | 7 | 3 | 0 | 2 | 1 | 0 | 0 | 1 |
| Dper | 40 | 15 | 8 | 5 | 3 | 1 | 1 | 1 | 0 | 2 | 1 | 1 |
| Dpse | 42 | 13 | 3 | 0 | 0 | 0 | 0 | 0 | 0 | 0 | 0 | 1 |
| Dsec | 613 | 243 | 149 | 55 | 25 | 14 | 8 | 9 | 8 | 4 | 1 | 1 |
| Dsim | 428 | 130 | 48 | 50 | 26 | 7 | 9 | 15 | 5 | 0 | 0 | 1 |
| Dvir | 118 | 30 | 14 | 3 | 1 | 3 | 0 | 0 | 0 | 2 | 0 | 1 |
| Dwil | 335 | 164 | 92 | 67 | 52 | 25 | 26 | 6 | 8 | 2 | 8 | 1 |
| Dyak | 316 | 218 | 182 | 174 | 185 | 177 | 166 | 110 | 79 | 46 | 25 | 1 |
| Nvit | 136 | 43 | 16 | 11 | 9 | 10 | 9 | 10 | 5 | 4 | 6 | 1 |
| Tcas | 25 | 8 | 14 | 7 | 8 | 7 | 5 | 4 | 7 | 1 | 1 | 1 |
| Aaeg | 114 | 39 | 20 | 17 | 9 | 6 | 7 | 4 | 11 | 6 | 2 | 2 |
| Agam | 186 | 71 | 45 | 42 | 29 | 23 | 25 | 10 | 11 | 5 | 5 | 2 |
| Amel | 426 | 176 | 93 | 48 | 36 | 23 | 23 | 14 | 4 | 4 | 0 | 2 |
| Apis | 266 | 100 | 58 | 28 | 26 | 15 | 6 | 5 | 3 | 5 | 3 | 2 |
| Bmor | 261 | 50 | 24 | 19 | 7 | 1 | 3 | 3 | 1 | 0 | 1 | 2 |
| Cqui | 200 | 64 | 29 | 10 | 10 | 4 | 1 | 0 | 1 | 1 | 1 | 2 |
| Dana | 1016 | 442 | 244 | 104 | 57 | 31 | 30 | 18 | 9 | 7 | 3 | 2 |
| Dere | 159 | 50 | 21 | 9 | 7 | 6 | 0 | 1 | 0 | 0 | 2 | 2 |
| Dgri | 1057 | 387 | 165 | 119 | 54 | 26 | 14 | 34 | 12 | 6 | 3 | 2 |
| Dmel | 547 | 267 | 76 | 30 | 16 | 4 | 6 | 1 | 3 | 1 | 0 | 2 |
| Dmoj | 547 | 197 | 61 | 26 | 7 | 2 | 3 | 1 | 1 | 1 | 1 | 2 |
| Dper | 95 | 32 | 12 | 4 | 2 | 1 | 0 | 0 | 1 | 0 | 1 | 2 |
| Dpse | 114 | 21 | 10 | 5 | 3 | 0 | 1 | 2 | 0 | 0 | 0 | 2 |
| Dsec | 731 | 340 | 176 | 83 | 39 | 18 | 21 | 13 | 7 | 8 | 5 | 2 |
| Dsim | 621 | 192 | 74 | 50 | 43 | 7 | 9 | 6 | 5 | 4 | 3 | 2 |
| Dvir | 247 | 73 | 25 | 15 | 2 | 3 | 1 | 0 | 1 | 0 | 0 | 2 |
| Dwil | 426 | 176 | 93 | 48 | 36 | 23 | 23 | 14 | 4 | 4 | 0 | 2 |
| Dyak | 143 | 95 | 81 | 71 | 61 | 67 | 53 | 56 | 42 | 30 | 27 | 2 |
| Nvit | 138 | 30 | 31 | 20 | 11 | 4 | 5 | 5 | 6 | 5 | 2 | 2 |
| Tcas | 24 | 7 | 4 | 9 | 3 | 6 | 2 | 2 | 4 | 4 | 5 | 2 |
| Aaeg | 0 | 30 | 14 | 17 | 5 | 12 | 4 | 2 | 3 | 0 | 2 | 4 |
| Agam | 0 | 31 | 36 | 22 | 20 | 15 | 16 | 7 | 5 | 5 | 1 | 4 |
| Amel | 16 | 159 | 113 | 69 | 44 | 22 | 23 | 12 | 12 | 4 | 5 | 4 |
| Apis | 13 | 146 | 63 | 33 | 15 | 7 | 4 | 5 | 3 | 2 | 1 | 4 |
| Bmor | 2 | 43 | 24 | 17 | 20 | 4 | 4 | 11 | 1 | 3 | 0 | 4 |
| Cqui | 0 | 27 | 41 | 22 | 14 | 11 | 2 | 1 | 1 | 3 | 0 | 4 |
| Dana | 3 | 254 | 236 | 199 | 85 | 45 | 35 | 17 | 9 | 3 | 2 | 4 |
| Dere | 2 | 35 | 26 | 17 | 11 | 4 | 2 | 5 | 2 | 1 | 1 | 4 |
| Dgri | 2 | 358 | 312 | 334 | 83 | 29 | 23 | 14 | 6 | 7 | 6 | 4 |
| Dmel | 0 | 89 | 75 | 48 | 19 | 20 | 4 | 2 | 0 | 2 | 0 | 4 |
| Dmoj | 1 | 80 | 58 | 53 | 21 | 9 | 4 | 1 | 1 | 1 | 0 | 4 |
| Dper | 2 | 32 | 32 | 17 | 4 | 5 | 4 | 1 | 2 | 1 | 1 | 4 |
| Dpse | 0 | 30 | 28 | 12 | 5 | 1 | 3 | 0 | 0 | 0 | 0 | 4 |
| Dsec | 0 | 169 | 163 | 117 | 59 | 39 | 25 | 15 | 6 | 5 | 3 | 4 |
| Dsim | 1 | 106 | 81 | 117 | 26 | 13 | 2 | 6 | 0 | 1 | 2 | 4 |
| Dvir | 2 | 49 | 46 | 61 | 14 | 5 | 8 | 1 | 0 | 0 | 0 | 4 |
| Dwil | 16 | 159 | 113 | 69 | 44 | 22 | 23 | 12 | 12 | 4 | 5 | 4 |
| Dyak | 2 | 19 | 18 | 14 | 15 | 10 | 10 | 8 | 6 | 8 | 3 | 4 |
| Nvit | 1 | 43 | 26 | 12 | 10 | 4 | 3 | 7 | 1 | 0 | 3 | 4 |
| Tcas | 3 | 9 | 8 | 6 | 3 | 6 | 5 | 2 | 4 | 2 | 1 | 4 |
| Aaeg | 0 | 0 | 0 | 3 | 2 | 0 | 6 | 4 | 3 | 1 | 3 | 6 |
| Agam | 0 | 0 | 1 | 2 | 12 | 4 | 7 | 3 | 3 | 2 | 3 | 6 |
| Amel | 0 | 0 | 2 | 25 | 18 | 20 | 14 | 14 | 15 | 4 | 3 | 6 |
| Apis | 0 | 0 | 0 | 7 | 26 | 12 | 8 | 2 | 4 | 3 | 0 | 6 |
| Bmor | 0 | 0 | 0 | 6 | 5 | 2 | 4 | 3 | 2 | 0 | 0 | 6 |
| Cqui | 0 | 0 | 0 | 3 | 8 | 7 | 3 | 4 | 1 | 1 | 0 | 6 |
| Dana | 0 | 0 | 0 | 15 | 33 | 42 | 38 | 14 | 12 | 9 | 4 | 6 |
| Dere | 0 | 0 | 3 | 2 | 12 | 7 | 1 | 2 | 2 | 1 | 0 | 6 |
| Dgri | 0 | 0 | 0 | 14 | 55 | 40 | 36 | 11 | 11 | 4 | 5 | 6 |
| Dmel | 0 | 0 | 0 | 1 | 7 | 7 | 7 | 4 | 3 | 6 | 1 | 6 |
| Dmoj | 0 | 0 | 0 | 4 | 7 | 11 | 6 | 0 | 2 | 1 | 1 | 6 |
| Dper | 0 | 0 | 0 | 4 | 2 | 6 | 4 | 3 | 1 | 0 | 0 | 6 |
| Dpse | 0 | 0 | 0 | 1 | 2 | 5 | 2 | 2 | 0 | 0 | 0 | 6 |
| Dsec | 0 | 0 | 0 | 7 | 31 | 31 | 19 | 13 | 14 | 3 | 8 | 6 |
| Dsim | 0 | 0 | 0 | 7 | 6 | 6 | 4 | 7 | 3 | 2 | 2 | 6 |
| Dvir | 0 | 0 | 0 | 3 | 13 | 8 | 6 | 5 | 5 | 1 | 0 | 6 |
| Dwil | 0 | 0 | 2 | 25 | 18 | 20 | 14 | 14 | 15 | 4 | 3 | 6 |
| Dyak | 0 | 0 | 0 | 0 | 3 | 3 | 4 | 0 | 3 | 4 | 3 | 6 |
| Nvit | 0 | 0 | 0 | 3 | 7 | 3 | 5 | 2 | 1 | 2 | 2 | 6 |
| Tcas | 0 | 0 | 0 | 2 | 3 | 4 | 0 | 2 | 0 | 0 | 1 | 6 |

The posterior t-test *p*-values of the pair-wise comparisons among the 20 insect species are as follows:

|  | 35 bp | 40 bp | 45 bp | 50 bp | 55 bp | 60 bp | 65 bp | 70 bp | 75 bp | 80 bp | 85 bp |
| --- | --- | --- | --- | --- | --- | --- | --- | --- | --- | --- | --- |
| 35 bp |  | 0.00019 | 1.74E-05 | 8.39E-06 | 1.3E-06 | 5.99E-07 | 3.95E-07 | 2.12E-07 | 1.77E-07 | 1.28E-07 | 1.19113E-07 |
| 40 bp | 0.000189697 |  | 4.65E-08 | 7.09E-08 | 1.75E-08 | 7.71E-09 | 3.07E-09 | 8.75E-10 | 7.08E-10 | 3.43E-10 | 3.04665E-10 |
| 45 bp | 1.73895E-05 | 4.65E-08 |  | 1.17E-05 | 1.11E-06 | 2.08E-07 | 5.19E-08 | 8.73E-09 | 5.69E-09 | 1.93E-09 | 1.69361E-09 |
| 50 bp | 8.3944E-06 | 7.09E-08 | 1.17E-05 |  | 0.001104 | 4.43E-05 | 8.74E-06 | 8.98E-07 | 4.21E-07 | 1.22E-07 | 9.69804E-08 |
| 55 bp | 1.29721E-06 | 1.75E-08 | 1.11E-06 | 0.001104 |  | 2.64E-08 | 5.15E-11 | 6.52E-11 | 9.19E-11 | 1.14E-10 | 2.72286E-10 |
| 60 bp | 5.99391E-07 | 7.71E-09 | 2.08E-07 | 4.43E-05 | 2.64E-08 |  | 1.86E-06 | 2.9E-07 | 7.4E-08 | 1.23E-07 | 3.5891E-07 |
| 65 bp | 3.94723E-07 | 3.07E-09 | 5.19E-08 | 8.74E-06 | 5.15E-11 | 1.86E-06 |  | 0.000433 | 9.69E-06 | 5.7E-06 | 1.14069E-05 |
| 70 bp | 2.12212E-07 | 8.75E-10 | 8.73E-09 | 8.98E-07 | 6.52E-11 | 2.9E-07 | 0.000433 |  | 0.000178 | 1.11E-05 | 3.29058E-05 |
| 75 bp | 1.76575E-07 | 7.08E-10 | 5.69E-09 | 4.21E-07 | 9.19E-11 | 7.4E-08 | 9.69E-06 | 0.000178 |  | 0.000149 | 0.000209062 |
| 80 bp | 1.28264E-07 | 3.43E-10 | 1.93E-09 | 1.22E-07 | 1.14E-10 | 1.23E-07 | 5.7E-06 | 1.11E-05 | 0.000149 |  | 0.030289033 |
| 85 bp | 1.19113E-07 | 3.05E-10 | 1.69E-09 | 9.7E-08 | 2.72E-10 | 3.59E-07 | 1.14E-05 | 3.29E-05 | 0.000209 | 0.030289 |  |

*B)* Non-insect species: genomic abundance

| Species | 35 bp | 40 bp | 45 bp | 50 bp | 55 bp | 60 bp | 65 bp | 70 bp | 75 bp | 80 bp | 85 bp | Number of mismatches |
| --- | --- | --- | --- | --- | --- | --- | --- | --- | --- | --- | --- | --- |
| Yeast | 2 | 2 | 3 | 0 | 0 | 0 | 0 | 0 | 0 | 0 | 0 | 1 |
| Roundworm | 19 | 3 | 6 | 4 | 0 | 1 | 2 | 2 | 1 | 0 | 1 | 1 |
| Mouse | 4802 | 3320 | 3045 | 2856 | 2497 | 1965 | 1433 | 1030 | 773 | 541 | 334 | 1 |
| Human | 2271 | 1116 | 772 | 528 | 403 | 224 | 107 | 87 | 51 | 44 | 22 | 1 |
| Yeast | 8 | 3 | 2 | 0 | 0 | 0 | 0 | 0 | 0 | 0 | 0 | 2 |
| Roundworm | 28 | 10 | 5 | 2 | 2 | 1 | 2 | 0 | 0 | 0 | 0 | 2 |
| Mouse | 4300 | 2346 | 1720 | 1343 | 1317 | 1193 | 921 | 714 | 553 | 441 | 286 | 2 |
| Human | 2841 | 1099 | 622 | 353 | 273 | 182 | 146 | 91 | 81 | 51 | 31 | 2 |
| Yeast | 0 | 1 | 3 | 0 | 4 | 0 | 0 | 0 | 0 | 0 | 0 | 4 |
| Roundworm | 0 | 4 | 5 | 6 | 3 | 3 | 1 | 4 | 0 | 2 | 0 | 4 |
| Mouse | 34 | 1444 | 1204 | 753 | 659 | 402 | 383 | 326 | 302 | 256 | 200 | 4 |
| Human | 28 | 840 | 633 | 361 | 214 | 114 | 106 | 60 | 65 | 44 | 46 | 4 |
| Yeast | 0 | 0 | 0 | 0 | 0 | 0 | 0 | 0 | 1 | 0 | 1 | 6 |
| Roundworm | 0 | 0 | 0 | 0 | 2 | 5 | 3 | 3 | 1 | 2 | 1 | 6 |
| Mouse | 0 | 0 | 2 | 146 | 391 | 317 | 288 | 191 | 180 | 157 | 123 | 6 |
| Human | 0 | 0 | 6 | 144 | 190 | 187 | 109 | 71 | 54 | 32 | 26 | 6 |

The posterior t-test *p*-values of the pair-wise comparisons among the non-insect species are as follows:

|  | 35 bp | 40 bp | 45 bp | 50 bp | 55 bp | 60 bp | 65 bp | 70 bp | 75 bp | 80 bp | 85 bp |
| --- | --- | --- | --- | --- | --- | --- | --- | --- | --- | --- | --- |
| 35 bp |  | 0.263931 | 0.149716 | 0.098801 | 0.093557 | 0.069741 | 0.064395 | 0.058607 | 0.058024 | 0.056273 | 0.055001 |
| 40 bp | 0.263931 |  | 0.018148 | 0.023802 | 0.031951 | 0.023238 | 0.022507 | 0.02164 | 0.023182 | 0.023899 | 0.024643 |
| 45 bp | 0.149716 | 0.018148 |  | 0.049155 | 0.076108 | 0.042348 | 0.037052 | 0.033873 | 0.035896 | 0.036601 | 0.037005 |
| 50 bp | 0.098801 | 0.023802 | 0.049155 |  | 0.287805 | 0.075108 | 0.063015 | 0.054755 | 0.057003 | 0.056802 | 0.055764 |
| 55 bp | 0.093557 | 0.031951 | 0.076108 | 0.287805 |  | 0.03053 | 0.04036 | 0.040482 | 0.045362 | 0.046792 | 0.047347 |
| 60 bp | 0.069741 | 0.023238 | 0.042348 | 0.075108 | 0.03053 |  | 0.074454 | 0.059404 | 0.065223 | 0.06416 | 0.062756 |
| 65 bp | 0.064395 | 0.022507 | 0.037052 | 0.063015 | 0.04036 | 0.074454 |  | 0.047919 | 0.06068 | 0.060588 | 0.059655 |
| 70 bp | 0.058607 | 0.02164 | 0.033873 | 0.054755 | 0.040482 | 0.059404 | 0.047919 |  | 0.092676 | 0.075285 | 0.069085 |
| 75 bp | 0.058024 | 0.023182 | 0.035896 | 0.057003 | 0.045362 | 0.065223 | 0.06068 | 0.092676 |  | 0.061926 | 0.059126 |
| 80 bp | 0.056273 | 0.023899 | 0.036601 | 0.056802 | 0.046792 | 0.06416 | 0.060588 | 0.075285 | 0.061926 |  | 0.060253 |
| 85 bp | 0.055001 | 0.024643 | 0.037005 | 0.055764 | 0.047347 | 0.062756 | 0.059655 | 0.069085 | 0.059126 | 0.060253 |  |
