# Supplementary material for: Motif mismatches in microsatellites: insights from genome-wide investigation among 20 insect species
Source: DNA Res. 2014 Nov 6;22(1):29–38. doi: 10.1093/dnares/dsu036 (PMC4379975; doi:10.1093/dnares/dsu036)
Supplement: Supplementary Data [file supp_dsu036_dsu036supp_table5.docx]

Supplementary Table 5. Genomic abundance of imperfect microsatellite loci based on length and number of mismatches in insect and non-insect species.

***A)* Insect genomes**

| Species | ≥ 30 bp SSR with ≥ 3 mismatches | ≥ 30 bp SSR with < 3 mismatches |
| --- | --- | --- |
| Aaeg | 3423 | 5109 |
| Agam | 5260 | 7763 |
| Amel | 10486 | 13047 |
| Apis | 6468 | 16270 |
| Bmor | 2619 | 4163 |
| Cqui | 4807 | 3825 |
| Dana | 3277 | 4316 |
| Dere | 2889 | 4067 |
| Dgri | 17857 | 23559 |
| Dmel | 4328 | 4123 |
| Dmoj | 20326 | 25544 |
| Dper | 7024 | 12851 |
| Dpse | 5131 | 10953 |
| Dsec | 2226 | 2088 |
| Dsim | 2006 | 2129 |
| Dvir | 15160 | 19497 |
| Dwil | 7731 | 14146 |
| Dyak | 3617 | 4353 |
| Nvit | 3121 | 14587 |
| Tcas | 1437 | 1381 |

***B)* Non-insect genomes**

| Species | ≥ 30 bp SSR with ≥ 3 mismatches | ≥ 30 bp SSR with < 3 mismatches |
| --- | --- | --- |
| Yeast | 133 | 213 |
| Roundworm | 883 | 1050 |
| Mouse | 164185 | 228621 |
| Human | 68066 | 81717 |
