# Supplementary material for: Motif mismatches in microsatellites: insights from genome-wide investigation among 20 insect species
Source: DNA Res. 2014 Nov 6;22(1):29–38. doi: 10.1093/dnares/dsu036 (PMC4379975; doi:10.1093/dnares/dsu036)
Supplement: Supplementary Data [file supp_dsu036_dsu036supp_table7.docx]

Supplementary Table 7. Number of imperfect microsatellites identified in chromosome X of different lines of *D. melanogaster*. The percentage values represent the proportion of imperfect microsatellites to the total number of microsatellites identified in the X chromosome of the individual lines.

| Line# | Imperfect SSRs | Percentage |
| --- | --- | --- |
| Line101 | 4108 | 31.6 |
| Line105 | 4071 | 31.3 |
| Line109 | 3682 | 30.7 |
| Line129 | 4233 | 31.5 |
| Line136 | 4199 | 31.7 |
| Line138 | 4349 | 31.8 |
| Line142 | 4221 | 32.0 |
| Line149 | 4088 | 31.4 |
| Line153 | 3709 | 31.6 |
| Line158 | 3824 | 31.3 |
| Line161 | 3959 | 31.4 |
| Line176 | 4399 | 32.0 |
| Line177 | 4469 | 32.3 |
| Line181 | 4287 | 31.6 |
| Line195 | 3999 | 31.1 |
| Line208 | 4292 | 31.6 |
| Line21 | 4135 | 31.6 |
| Line217 | 4028 | 31.5 |
| Line227 | 4200 | 31.8 |
| Line228 | 3940 | 31.0 |
| Line229 | 3910 | 31.3 |
| Line233 | 3963 | 31.2 |
| Line235 | 4041 | 31.4 |
| Line237 | 3991 | 31.5 |
| Line239 | 4026 | 31.1 |
| Line256 | 3964 | 31.0 |
| Line26 | 4204 | 31.6 |
| Line272 | 4068 | 31.7 |
| Line280 | 4087 | 31.4 |
