# Supplementary material for: Motif mismatches in microsatellites: insights from genome-wide investigation among 20 insect species
Source: DNA Res. 2014 Nov 6;22(1):29–38. doi: 10.1093/dnares/dsu036 (PMC4379975; doi:10.1093/dnares/dsu036)
Supplement: Supplementary Data [file supp_dsu036_dsu036supp_table8.docx]

Supplementary Table 8. Polymorphisms in the extended chromosomal region (1 kb both ends of microsatellites) among *D. melanogaster*. The alleles of SNPs (shown in column 1) identified within microsatellites show differential association with polymorphisms in the flanking sequences.

| Locus and Alleles | n | m | k | fixed |
| --- | --- | --- | --- | --- |
| Chr X: 8114522..8116564 | 24 | 31 | 11.018 | 4 |
| rs204392359 Allele-A | 15 | 24 | 7.924 |  |
| rs204392359 Allele-T | 9 | 20 | 5.611 |  |
| Chr X: 972789..974820 | 29 | 14 | 5.389 | 3 |
| rs204306547 Allele-C | 13 | 10 | 3.077 |  |
| rs204306547 Allele-T | 16 | 2 | 0.358 |  |
| Chr X: 8323599..8325631 | 11 | 15 | 4.364 | 2 |
| rs205546548 Allele-A | 6 | 11 | 4.067 |  |
| rs205546548 Allele-C | 5 | 6 | 2.6 |  |

n= Number of sequences; m= Total number of variable sites k= Average number of nucleotide differences; fixed=fixed mutations between the two alleles.
